# Data on the value of elevated circulating mimecan levels for detecting poor coronary collateralization in patients with stable angina and chronic total occlusion

**DOI:** 10.1016/j.dib.2016.09.030

**Published:** 2016-09-22

**Authors:** Ying Shen, Feng Hua Ding, Rui Yan Zhang, Qi Zhang, Lin Lu, Wei Feng Shen

**Affiliations:** a Department of Cardiology, Rui Jin Hospital, Shanghai Jiaotong University School of Medicine, Shanghai 200025, People׳s Republic of China; b Institute of Cardiovascular Diseases, Shanghai Jiaotong University School of Medicine, Shanghai 200025, People׳s Republic of China

## Abstract

The data presented here support the research article “Association of serum mimecan with angiographic coronary collateralization in patients with stable coronary artery disease and chronic total occlusion” (Shen et al., 2016) [1] where elevated circulating mimecan levels reflected poor angiographic coronary collateralization in such patients. The data included in this article are composed by one figure and consist of (1) validation of serum mimecan measurement by assessing inter- and intra-assay variability in 45 samples; (2) findings on the relation of clinical and angiographic characteristics and biochemical parameters to coronary collateralization in 559 patients; (3) the diagnostic value of serum mimecan for poor collateralization, which was derived from plotting receiver-operating characteristic curves and logistic regression analysis.

**Specifications Table**

**Table:** 

Subject area	Clinical research
More specific subject area	Cardiology
Type of data	Figure
How data was acquired	Register database
Data format	Raw, analyzed
Experimental factors	Determination of clinical, angiographic and biochemical parameters
Experimental features	Association between serum mimecan and coronary collateralization was assessed in 559 patients with stable angina and chronic total occlusion
Data source location	People׳s Republic of China
Data accessibility	Data are within this article

**Value of the data**

• Data on low coronary collateralization in stable angina patients who have concurrently elevated circulating mimecan levels are suited for studies investigating the mechanism of collateral development.
• The data provide a novel biomarker of poor collateral formation which may be instrumental in further studies on risk stratification and cardiovascular outcome particularly for patients with chronic coronary total occlusion.
• The multivariable data involving clinical, biochemical, and angiographic parameters provide opportunities for the application of advanced approaches in an integrity way, potentially leading to new insight on the pathophysiology of coronary artery disease.

## 1 Data

The data consist of clinical characteristics and angiographic features along with biochemical measurements associated with coronary collateralization in patients with stable angina and chronic total occlusion, and the results of multivariable logistic regression analysis for diagnostic value of serum mimecan in detecting poor collateralization. The data also include the coefficient of variance for serum mimecan assay [1].

## 2 Experimental design, material and methods

### 2.1 Study population

**Fig. 1:**
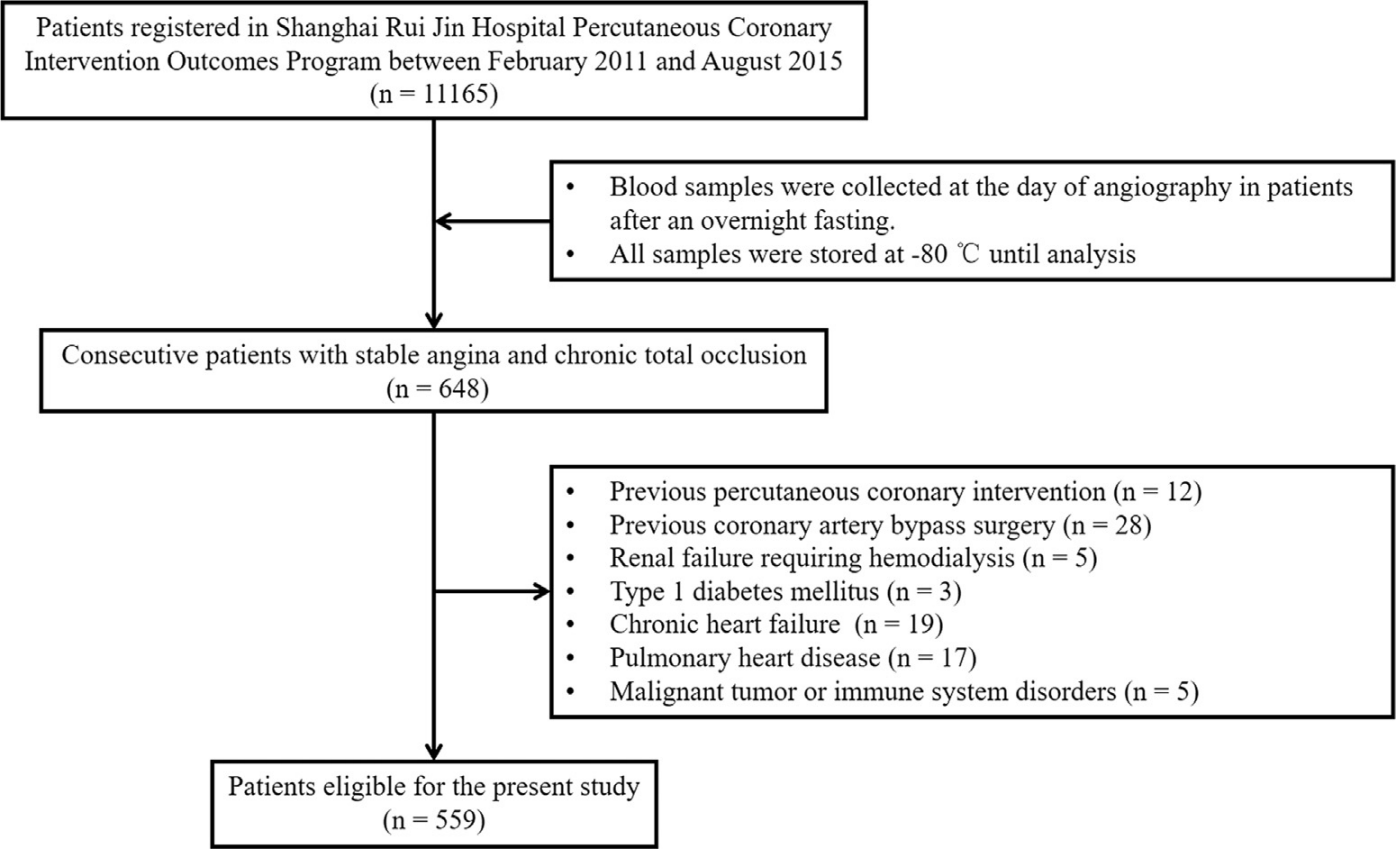
Flowchart of patient enrollment.

The data included a cohort of 648 consecutive patients with stable angina and chronic total occlusion (>3 months) of at least one major epicardial coronary artery. The participants were prospectively entered a database [2]. Information on patient demographics, clinical and angiographic feature and in-hospital management was collected retrospectively, whereas clinical outcome during follow-up was identified prospectively. For the purpose of the study that the data are based on, 89 patients were excluded by the exclusion criteria, and 559 patients were eligible for final analysis (Fig. 1).

### 2.2 Collateral grading

Coronary angiography films were reviewed by two experienced cardiologists blinded to the clinical and demographic data of all patients. Any differences in interpretation were resolved by a third reviewer who was blinded to the reading of the first two reviewers. The presence and significance of collaterals filling from the contra-lateral vessel were graded on the Rentrop scoring system according to visibility and filling characteristics [3]. Rentrop score of 0 and 1 was classified as poor collateralization and Rentrop score of 2 and 3 as good collateralization [4], [5].

### 2.3 Mimecan measurement

Blood samples taken at the day of angiography were transferred immediately into pyrogen-free tubes containing EDTA-2Na (1 mg/ml) and then centrifuged immediately at 1500*g* for 15 min. The resulting plasma samples were stored frozen at −80 °C in multiple aliquots until analysis. A commercially available ELISA kit (Antibodies-online Inc., Atlanta, GA, USA) was used for determination of mimecan levels. To assess the accuracy of serum mimecan measurement, inter-assay variability was made by calculating the coefficient of variance (CV=standard deviation/mean×100) of 4 replicate measurements of each 15 samples from tertiles of mimecan, and intra-assay variability was assessed by calculating the CV of 2 replicate for 45 samples.

### 2.4 Statistical analysis

All analyses were performed with the SPSS 20.0 for Windows (SPSS, Inc., Chicago, IL, USA). Continuous and categorical data are presented as mean and standard deviation (SD) or median (interquartile range [IQR]) and frequencies or percentages, and statistical difference between groups was evaluated with the student *t*-test and chi-square test, respectively. Correlation between serum mimecan and Rentrop scores was determined by the Spearman׳s rho test. Multivariable logistic regression analysis was performed to assess the independent determinants of poor collateralization. In model 1, conventional clinical, biochemical and angiographic variables were include, and in model 2, serum level of mimecan (per SD) was additionally included as well as the variables in model 1. Receiver-operating characteristic curves were plotted with the predicted probabilities for poor collateralization derived from logistic regression models. The C statistics were compared using DeLong method, and net reclassification improvement (NRI) and integrated discrimination improvement (IDI) of the addition of mimecan in model 2 were calculated to assess predictive performance improvement [6]. A p<0.05 was considered to be statistically significant.
